# Undenatured type II collagen mitigates inflammation and cartilage degeneration in healthy Labrador Retrievers during an exercise regimen

**DOI:** 10.1093/tas/txab084

**Published:** 2021-05-10

**Authors:** J L Varney, J W Fowler, C N Coon

**Affiliations:** Four Rivers Kennel, LLC, Walker, MO 64790, USA

**Keywords:** biomarkers, dog, exercise, inflammation, Labrador Retriever, undenatured type II collagen

## Abstract

The aim of this experiment was to evaluate the effect of undenatured type II collagen supplementation on inflammation and cartilage degeneration after exercise in healthy dogs. Forty healthy Labrador Retrievers (20 male/20 female; range 5-12 yr; average 8 yr) were sorted into two groups: undenatured type II collagen group receiving 40 mg UC-II (10 mg collagen type II/min. 3% undenatured type II collagen; Lonza Consumer Health, Inc.) and placebo group receiving 40 mg maltodextrin daily by capsule. After 2-wk loading, all dogs began an 11-wk endurance exercise regimen consisting of two weekly runs, starting at 5 km and increasing incrementally to 8 km, with one final 16 km run. Blood samples were collected at baseline, pre and post first 5 km run, and pre- and post-16 km run. Activity per kilometer was greater in male undenatured type II collagen vs. male placebo over all runs (*P* = 0.004), and average moving speed was greater in all undenatured type II collagen dogs compared with placebo over all runs (*P* < 0.001). Hematology analysis indicated that during the first insult, undenatured type II collagen dogs had a greater lymphocyte count (*P* < 0.001) and lymphocyte percentage (*P* = 0.001) vs. placebo dogs. Undenatured type II collagen dogs had a lesser neutrophil percentage (*P* = 0.042) and neutrophil to lymphocyte ratios (*P* = 0.001) compared to placebo dogs. For the final insult, undenatured type II collagen dogs had greater lymphocyte percentage (*P* = 0.013) and lesser mean corpuscular hemoglobin concentration (*P* = 0.043) compared with placebo dogs. Both groups had significant changes between timepoints for several hematological parameters. Biomarker IL-6 was lesser in undenatured type II collagen dogs compared with placebo at post 5 km (*P* = 0.037). Cartilage oligomeric matrix protein (COMP) was lesser in undenatured type II collagen dogs at post 16 km (*P* = 0.023), and only the placebo dogs had a significant increase in COMP from pre to post 16 km (*P* = 0.021). In summary, Labrador Retrievers supplemented with undenatured type II collagen had decreased inflammation and cartilage degeneration compared with nonsupplemented dogs during exercise.

## INTRODUCTION

Undenatured type II collagen is a supplement derived from chicken sternum cartilage that has demonstrated promising results in the management of osteoarthritis and reduction of lameness in both human (Crowley et al., 2009; Lugo et al., 2013) and animal trials (D’Altilio et al., 2007) (Gencoglu et al., 2020). Current over-the-counter therapies for the treatment of lameness and osteoarthritis include nutraceuticals such as glucosamine hydrochloride, chondroitin sulfate, hyaluronic acid, and methylsulfonylmethane with varying degrees of efficacy (Vandeweerd et al., 2012; Scott et al., 2017). Veterinarian prescribed therapies include non-steroidal anti-inflammatory drugs (NSAIDs) and injections of polysulfated glycosaminoglycan which have the potential of causing deleterious side effects (Henrotin et al., 2005). Investigating alternative and multifaceted therapies for the management of lameness is necessary to improve quality of life in companion animals.

Previous experiments have evaluated the efficacy of undenatured type II collagen in known arthritic dogs (Deparle et al., 2005) and in dogs with temporary mobility impairment (Stabile et al., 2019). In order to evaluate the efficacy and safety of undenatured type II collagen in healthy Labrador Retrievers, this experiment used an endurance exercise model to induce inflammation and cartilage turnover. Inflammatory biomarkers (neutrophil: lymphocyte ratio and interleukin-6), muscle breakdown biomarkers (creatine phosphokinase), and cartilage degradation biomarkers (cartilage oligomeric matrix protein) were used to compare the effect of undenatured type II collagen supplemented dogs versus maltodextrin placebo supplemented dogs.

## MATERIALS AND METHODS

All animal care and procedures were reviewed and approved by the Institutional Care and Use Committee at Four Rivers Kennel, LLC under protocol FRK-22.

### Animals and Housing

Forty Labrador Retrievers (20 male/20 female) were used in this experiment and averaged 8 yr of age (range: 5–12 yr). All dogs were housed in individual kennels overnight and allowed free access to outside airing yards for six to eight hours daily, weather permitting. All dogs had ad libitum access to automatic waterers inside and outside. All dogs were fed once daily in the morning as per their treatment requirements. Prophylactic heartworm prevention containing ivermectin and pyrantel (Heartgard Plus; Boehringer Ingelheim Animal Health USA; Duluth, GA) was administered monthly.

### Diet and Treatments

All Labrador Retrievers were fed a poultry and corn based kennel diet, MFA Gold N Pro (protein 27%; fat 15%; fiber 3%; moisture 10%; energy content 3.663 kcal/g) (Missouri Farmers Association, Inc.; Columbia, MO) for the duration of the experiment. Feed amounts were determined based on historical data to maintain starting body weight. Feed consumption was determined daily by weighing feed provided and feed refusals.

Each dog was sorted to one of two equalized treatment groups based on age, sex, and bodyweight. Related dogs were sorted equally between the treatment groups. Undenatured type II collagen group received 40 mg UC-II (10 mg collagen type II/min. 3% undenatured type II collagen; Lonza Consumer Health, Inc.; Morristown, NJ) daily and placebo group received 40 mg maltodextrin daily. Both treatments were given in capsule form by mouth, once daily in the morning.

### Running Exercise

After 2 wk of supplement loading, all dogs began a twice-weekly running regimen. The regimen was as follows: Week 1–2, loading; Week 3–5, 2× 5 km runs; Week 6–8, 2× 6.5 km runs; Week 9–11, 2× 8 km runs; Week 12, 2× 3 km runs; Week 13, 1× 16 km run. The first 5 km run and the final 16 km run were used as the interest points for the biggest exercise insult to the dogs. All dogs ran alongside an all-terrain vehicle in the bush where they were free to run, swim, play, etc. but met the minimum prescribed distance. All dogs wore Actical accelerometer collars (Starr Life Sciences Corp; Oakmont, PA) to quantify activity intensity and global positioning collars (GPS) (Garmin Intl; Olathe, KS) to determine actual distance ran and average moving speed. Accelerometer data were divided by distance run to obtain an activity per kilometer (APKm) value.

### Sample Collection and Laboratory Analysis

Blood samples were taken from each dog via jugular venipuncture prior to loading (baseline), 1 h prior to the first 5 km run (pre-5 km), 24 h after the first 5 km run (post-5 km), 30 min prior to the final 16 km run (pre-16 km), and 24 h after the final 16 km run (post-16 km). For biomarker analysis, blood was collected into serum separator tubes (BD Lifesciences, San Jose, CA), allowed to clot for 60 min at room temperature, centrifuged at 1500 × *g* for 15 min, serum aliquoted, and frozen at −80 °C. For hematologic purposes, whole blood was collected into EDTA vacutainers (BD Lifesciences, San Jose, CA) and analyzed immediately via an in-house automated analyzer (Abaxis HM5; Abaxis Global Diagnostics, Union City, CA).

Commercial biomarker kits were used for the analysis of interleukin-6 (#AB193686; Abcam; Burlingame, CA) (IL-6), creatine kinase-MM (#AB197749; Abcam; Burlingame, CA) (CKM), and cartilage oligomeric matrix protein (#MBS2123988; MyBiosource; San Diego, CA) (COMP). All samples were analyzed in duplicate. Linear regression equations were developed from standard curves on each plate and used to calculate concentrations in each sample.

### Statistical Analysis

JMP 14.1.0 (SAS Institute Inc., Cary, NC) was used to create a mixed model for biomarkers and hematological values to compare treatment groups by sex and timepoint. “Dog” was analyzed as the random effect. If the mixed model indicated significant difference, a post-hoc Tukey’s test was applied and used to determine significantly different means. Sex was analyzed as a fixed effect due potential differences in body composition and metabolism. Results were considered significant at *P*-value <0.05. Results are presented as mean ± standard error.

## RESULTS

### Body Weights

Overall, body weights were not significantly different between groups (*P* = 0.369) (Table 1). Undenatured type II collagen males had lower body weights compared with placebo males (*P* = 0.023), but females had no significant differences (*P* = 0.375). Body weights between treatments were not different at any specific week between treatments for overall, males, or females, which indicates that the male difference is not likely clinically relevant.

**Table 1.** Body weight (kg) comparison between undenatured type II collagen (UC-II) dogs and placebo dogs, both overall and between beginning and end weeks

				*P*-value		
Weeks	Treatment	UC-II	Placebo	Treatment	Week	Treatment × Week
Overall	All	28.77 ± 0.22	29.12 ± 0.22	0.369	0.395	0.999
Overall	Male	31.16 ± 0.33	32.23 ± 0.33	0.023	0.651	0.999
Overall	Female	26.39 ± 0.30	26.01 ± 0.30	0.375	0.605	0.999

### Feed Intake

Overall, both feed offered and feed intake was not significantly different between groups (*P* = 0.572) (Table 2). Feed intake was not significantly different between weeks or by treatment and week. Feed intake was not significantly different between treatments for both males (*P* = 0.134) and females (*P* = 0.642).

**Table 2.** Feed offered and feed consumption comparison between undenatured type II collagen (UC-II) dogs and placebo dogs

				*P*-value		
Parameter	Treatment	UCII	Placebo	Treatment	Week	Treatment × Week
Feed offered (g)	Overall	565 ± 16	575 ± 16	0.655	0.999	0.999
	Male	610 ± 23	655 ± 23	0.176	0.999	0.999
	Female	520 ± 21	495 ± 21	0.408	0.999	0.999
Feed consumption (g)	Overall	562 ± 20	578 ± 20	0.572	0.996	0.999
	Male	605 ± 21	652 ± 21	0.164	0.767	0.958
	Female	520 ± 22	505 ± 22	0.642	0.999	0.999

### Activity and Moving Speed

Actual distance ran was not different between treatments over all runs, with undenatured type II collagen dogs running an average distance of 6.7 ± 0.13 km for all runs and placebo dogs running an average distance of 6.68 ± 0.13 for all runs (*P* = 0.548).

For all runs, APKm was not different between treatment overall (*P* = 0.459). Undenatured type II collagen males had greater activity at 45576 APKm compared with placebo males at 43701 APKm (*P* = 0.004) (Table 3). No difference in APKm was found between female groups (*P* = 0.196).

**Table 3.** Comparison of activity per kilometer and average moving speeds over all runs for undenatured type II collagen (UC-II) dogs and placebo dogs

				*P*-value		
Parameter	Sex	UC-II	Placebo	Treatment	Run	Treatment × run
Activity per kilometer	Overall	44019 ± 370	43633 ± 372	0.459	0.012	0.709
	Male	45576 ± 470	43701 ± 471	0.004	0.019	0.492
	Female	42656 ± 578	43715 ± 579	0.196	0.552	0.982
Average moving speed (kph)	Overall	10.73 ± 0.05	10.43 ± 0.05	<0.001	<0.001	0.994
	Male	10.94 ± 0.06	10.85 ± 0.06	0.268	<0.001	0.993
	Female	10.52 ± 0.08	10.02 ± 0.08	<0.001	0.001	0.999

Average moving speed was greater in the undenatured type II collagen group compared with the placebo group (*P* < 0.001) (Table 3). This was primarily driven by the females (*P* < 0.001). Moving speed was significantly different between run distances for both treatment groups (*P* < 0.001).

### Hematology

For the first insult (Table 4), when compared with placebo, undenatured type II collagen dogs had a greater lymphocyte count (*P* < 0.001) and lymphocyte percentage (*P* = 0.001). Undenatured type II collagen dogs had lesser neutrophil percentage (*P* = 0.042) and neutrophil to lymphocyte ratios (*P* = 0.001) compared to placebo dogs.

**Table 4.** Hematology results between undenatured type II collagen (UC-II) and placebo treatments during the first insult at timepoints (TP) baseline, pre first 5 km run (pre 5 km), and post first 5 km run (post 5 km)

		UC-II			Placebo				*P*-value		
Variable	Sex	Baseline	Pre 5 km	Post 5 km	Baseline	Pre 5 km	Post5 km	SEM	Trx	TP	Trx × TP
White blood cells, 10^9^/l	MF	9.43^b^	10.88^ab^	11.61^a^	9.25^b^	11.24^a^	10.86^a^	0.18	0.554	0.000	0.383
	M	9.00^b^	10.88^ab^	11.63^a^	9.86^b^	12.00^a^	11.86^a^	0.27	0.131	0.000	0.738
	F	9.87	10.88	11.59	8.63	10.48	9.86	0.23	0.007	0.005	0.406
Lymphocytes, 10^9^/l	MF	1.64	1.73	1.69	1.49	1.43	1.41	0.04	0.000	0.937	0.624
	M	1.58	1.68	1.68	1.73	1.55	1.43	0.04	0.392	0.645	0.164
	F	1.71	1.79	1.70	1.25	1.31	1.39	0.06	0.000	0.795	0.770
Monocytes, 10^9^/l	MF	0.45	0.52	0.53	0.41^b^	0.58^a^	0.54^ab^	0.02	0.886	0.023	0.572
	M	0.40	0.48	0.56	0.36	0.60	0.55	0.03	0.655	0.024	0.469
	F	0.51	0.57	0.51	0.45	0.56	0.53	0.03	0.809	0.465	0.835
Neutrophils, 10^9^/l	MF	7.17^b^	8.38^ab^	9.25^a^	7.12^b^	8.99^a^	8.69^a^	0.16	0.995	0.000	0.269
	M	6.90^b^	8.47^ab^	9.25^a^	7.47^b^	9.51^a^	9.65^a^	0.26	0.168	0.001	0.815
	F	7.44	8.30	9.25	6.77	8.47	7.79	0.19	0.062	0.002	0.164
Eosinophils, 10^9^/l	MF	0.12^ab^	0.17^a^	0.09^b^	0.16	0.18	0.16	0.01	0.088	0.236	0.661
	M	0.09	0.19	0.10	0.22	0.26	0.22	0.02	0.011	0.288	0.865
	F	0.15	0.15	0.08	0.11	0.10	0.10	0.01	0.296	0.241	0.416
Basophils, 10^9^/l	MF	0.05^ab^	0.07^a^	0.04^b^	0.07	0.07	0.06	0.01	0.257	0.161	0.248
	M	0.03^b^	0.08^a^	0.04^ab^	0.08	0.08	0.08	0.01	0.004	0.153	0.288
	F	0.07	0.07	0.05	0.06	0.05	0.04	0.01	0.177	0.304	0.641
Lymphocytes, %	MF	17.84^a^	16.11^ab^	14.85^b^	16.02^a^	12.93^b^	13.28^ab^	0.36	0.001	0.001	0.578
	M	17.83	15.74	15.00	17.75	13.33	12.44	0.55	0.097	0.004	0.527
	F	17.84	16.47	14.70	14.28	12.53	14.11	0.48	0.004	0.248	0.257
Monocytes, %	MF	4.65	4.89	4.58	4.11	5.14	5.04	0.17	0.862	0.295	0.437
	M	4.35	4.44	4.73	3.57	4.92	4.72	0.22	0.818	0.300	0.514
	F	4.95	5.33	4.42	4.65	5.35	5.36	0.25	0.665	0.647	0.584
Neutrophils, %	MF	75.65^b^	76.83^ab^	79.45^a^	77.06	79.79	79.60	0.63	0.042	0.002	0.299
	M	76.46	77.39	79.06	69.54	78.82	80.21	0.57	0.620	0.034	0.642
	F	74.83	76.27	79.83	78.58	80.76	78.99	0.53	0.013	0.075	0.059
Eosinophils, %	MF	1.31^ab^	1.54^a^	0.78^b^	1.82	1.56	1.46	0.11	0.077	0.190	0.467
	M	1.00	1.76	0.87	2.32	2.19	1.91	0.20	0.017	0.456	0.621
	F	1.62	1.31	0.68	1.32	0.92	1.00	0.11	0.572	0.068	0.354
Basophils, %	MF	0.55^ab^	0.67^a^	0.35 ^b^	0.73	0.58	0.64	0.04	0.095	0.200	0.093
	M	0.36^ab^	0.68^a^	0.32^b^	0.82	0.70	0.70	0.05	0.005	0.321	0.151
	F	0.74	0.66	0.38	0.64	0.45	0.57	0.05	0.710	0.262	0.297
Red blood cells, 10^12^/l	MF	8.07	8.19	8.10	8.20	8.30	8.38	0.05	0.082	0.596	0.840
	M	8.54	8.62	8.42	8.20	8.33	8.35	0.06	0.069	0.754	0.644
	F	7.60	7.75	7.78	8.21	8.36	8.41	0.08	0.000	0.558	0.997
Hemoglobin, g/dl	MF	16.59	17.11	17.1	16.67	17.23	17.24	0.11	0.605	0.074	0.993
	M	17.55	17.94	17.57	16.67	17.15	17.29	0.12	0.008	0.298	0.533
	F	15.62	16.28	16.62	16.66	17.31	17.19	0.17	0.010	0.130	0.800
Hematocrit, %	MF	56.9	57.45	57.11	57.47	58.48	58.62	0.35	0.140	0.610	0.858
	M	60.05	59.81	58.80	57.52	58.00	57.80	0.42	0.038	0.821	0.755
	F	53.76	55.09	55.43	57.41	58.97	59.44	0.53	0.000	0.249	0.988
Mean corpuscular volume, fl	MF	70.45	70.30	70.60	70.10	70.10	69.90	0.25	0.409	0.992	0.917
	M	70.20	69.50	69.90	70.10	69.60	69.10	0.30	0.664	0.628	0.819
	F	70.70	71.10	71.30	70.10	70.60	70.70	0.39	0.484	0.818	0.998
Mean corpuscular hemoglobin, pg	MF	20.56	20.91	21.17	20.32^b^	20.67^ab^	20.95^a^	0.08	0.066	0.001	0.996
	M	20.58	20.80	20.93	20.34	20.61	20.71	0.08	0.165	0.160	0.991
	F	20.54	21.01	21.40	20.30	20.72	20.53	0.11	0.233	0.003	0.979
Mean corpuscular hemoglobin concentration, g/dl	MF	29.14^b^	29.78^ab^	29.95^a^	28.99^b^	29.43^ab^	29.99^a^	0.11	0.300	0.000	0.562
	M	29.26	29.97	29.92	28.98^b^	29.52^ab^	29.92^a^	0.11	0.226	0.005	0.649
	F	29.02	29.58	29.98	29.00	29.33	29.05	0.12	0.788	0.003	0.825
Red blood cell distribution width, %	MF	16.26	16.23	16.08	16.13	16.17	16.12	0.07	0.712	0.826	0.896
	M	16.29	16.70	16.35	16.00	16.22	16.22	0.09	0.119	0.402	0.753
	F	16.23	15.76	15.81	16.25	16.11	16.01	0.11	0.397	0.409	0.833
Platelets, 10^9^/l	MF	351.45	367	361.2	361.1	358.4	385.75	8.08	0.604	0.688	0.711
	M	320.90	329.40	309.00	313.10	314.60	328.10	9.35	0.952	0.977	0.755
	F	382.00	404.60	413.40	409.10	402.20	443.40	10.36	0.391	0.419	0.787
Mean platelet volume, fl	MF	9.68	9.63	9.61	9.69	9.41	9.73	0.07	0.820	0.533	0.550
	M	9.67	9.75	9.72	9.70	9.64	10.11	0.09	0.583	0.530	0.535
	F	9.68	9.51	9.49	9.68	9.17	9.34	0.09	0.368	0.278	0.743
Plateletcrit, %	MF	0.34	0.35	0.35	0.35	0.34	0.37	0.01	0.668	0.649	0.553
	M	0.31	0.32	0.30	0.30	0.30	0.33	0.01	0.882	0.920	0.582
	F	0.37	0.38	0.39	0.40	0.37	0.41	0.01	0.614	0.550	0.684
Platelet distribution width, %	MF	36.71	36.58	36.27	36.51	36.23	36.62	0.15	0.830	0.839	0.614
	M	36.84	36.89	36.32	37.02	36.81	37.45	0.22	0.359	0.989	0.507
	F	36.58	36.26	36.22	36.00	35.65	35.79	0.19	0.175	0.756	0.980
Neutrophil to Lymphocyte Ratio	MF	4.56	4.99	5.68	5.02^b^	6.75^a^	6.69^a^	0.17	0.001	0.001	0.252
	M	4.54	5.26	5.67	4.32^b^	6.55^ab^	7.26^a^	0.26	0.072	0.003	0.269
	F	4.58	4.73	5.68	5.71	6.95	6.15	0.23	0.004	0.275	0.249

^abc^ Within rows and treatment, means without a common superscript are significantly different (P<0.05).

For the final insult (Table 5), undenatured type II collagen dogs had greater lymphocyte percentage (*P* = 0.013) and lesser mean corpuscular hemoglobin concentration (*P* = 0.043) compared with placebo dogs.

**Table 5.** Hematology results between undenatured type II collagen (UC-II) and placebo treatment (Trx) groups during the final insult at timepoints (TP) pre 16 km run and post 16 km run

		UC-II		Placebo			*P*-value		
Variable	Sex	Pre 16 km	Post 16 km	Pre 16 km	Post 16 km	SEM	Trx	TP	Trx × TP
White blood cells, 10^9^/l	MF	10.23	11.23	10.62	11.50	0.25	0.500	0.057	0.894
	M	10.41	11.12	12.01	12.41	0.35	0.957	0.039	0.817
	F	10.02	11.36	9.23	10.59	0.32	0.216	0.037	0.986
Lymphocytes, 10^9^/l	MF	1.55	1.80	1.43	1.63	0.05	0.125	0.019	0.776
	M	1.69	1.70	1.54	1.64	0.07	0.429	0.413	0.734
	F	1.39	1.91	1.32	1.62	0.07	0.161	0.002	0.366
Monocytes, 10^9^/l	MF	0.40	0.52	0.43	0.55	0.02	0.578	0.016	0.935
	M	0.37	0.56	0.46	0.60	0.03	0.314	0.690	0.725
	F	0.44	0.48	0.39	0.50	0.03	0.843	0.334	0.618
Neutrophils, 10^9^/l	MF	8.04	8.71	8.48	9.03	0.21	0.368	0.149	0.884
	M	8.06	8.64	9.65	9.80	0.31	0.026	0.019	0.723
	F	8.02	8.79	7.31	8.26	0.26	0.238	0.105	0.873
Eosinophils, 10^9^/l	MF	0.17	0.15	0.17	0.16	0.01	0.805	0.582	0.790
	M	0.21	0.16	0.21	0.17	0.02	0.973	0.543	0.852
	F	0.06	0.05	0.06	0.06	0.02	0.634	0.688	0.916
Basophils, 10^9^/l	MF	0.06	0.06	0.08	0.07	0.01	0.244	0.767	0.988
	M	0.07	0.07	0.09	0.08	0.01	0.241	0.270	0.901
	F	0.06	0.05	0.06	0.06	0.01	0.618	0.917	0.917
Lymphocytes, %	MF	15.34	16.48	13.55	14.57	0.38	0.013	0.144	0.936
	M	16.76	16.14	12.78	13.42	0.55	0.002	0.756	0.526
	F	13.77	16.86	14.31	15.71	0.53	0.771	0.035	0.416
Monocytes, %	MF	3.93	4.59	4.15	4.63	0.19	0.733	0.134	0.815
	M	3.40	4.99	3.86	4.79	0.27	0.802	0.992	0.525
	F	4.51	4.14	4.43	4.47	0.27	0.828	0.771	0.718
Neutrophils, %	MF	78.44	77.48	79.43	78.24	0.44	0.326	0.227	0.893
	M	77.07	77.63	79.69	78.69	0.69	0.194	0.019	0.578
	F	79.96	77.32	79.16	77.78	0.54	0.876	0.071	0.564
Eosinophils, %	MF	1.68	1.35	1.57	1.36	0.13	0.838	0.297	0.806
	M	2.12	1.51	1.73	1.31	0.20	0.464	0.875	0.814
	F	1.20	1.18	1.42	1.41	0.16	0.492	0.961	0.985
Basophils, %	MF	0.63	0.54	0.72	0.63	0.05	0.379	0.400	0.986
	M	0.68	0.63	0.77	0.67	0.07	0.647	0.200	0.860
	F	0.57	0.45	0.66	0.59	0.07	0.417	0.515	0.870
Red blood cells, 10^12^/l	MF	8.45	8.00	8.35	7.89	0.07	0.412	0.000	0.976
	M	8.65	7.91	8.46	7.76	0.10	0.310	0.598	0.917
	F	8.23	8.10	8.25	8.03	0.09	0.881	0.337	0.820
Hemoglobin, g/dl	MF	17.75	16.95	17.68	16.71	0.14	0.549	0.001	0.740
	M	18.08	16.68	17.82	16.29	0.20	0.348	0.000	0.857
	F	17.38	17.24	17.54	17.12	0.18	0.961	0.471	0.708
Hematocrit, %	MF	59.91	56.5	58.63	55.01	0.48	0.118	0.000	0.905
	M	61.40	55.62	59.43	53.83	0.74	0.120	0.000	0.939
	F	58.25	57.48	57.83	56.19	0.61	0.496	0.342	0.732
Mean corpuscular volume, fl	MF	70.89	70.68	70.25	69.6	0.31	0.164	0.486	0.722
	M	71.00	70.40	70.30	69.30	0.36	0.219	0.000	0.783
	F	70.78	71.00	70.20	69.90	0.51	0.429	0.971	0.805
Mean corpuscular hemoglobin, pg	MF	21.07	21.21	21.17	21.19	0.09	0.838	0.703	0.759
	M	20.91	21.08	21.05	21.02	0.12	0.874	0.274	0.692
	F	21.24	21.34	21.29	21.35	0.15	0.936	0.802	0.950
Mean corpuscular hemoglobin concentration, g/dl	MF	29.71	30.01	30.14	30.42	0.10	0.043	0.152	0.894
	M	29.43	29.96	29.95	30.31	0.14	0.129	0.781	0.817
	F	30.01	30.07	30.32	30.52	0.15	0.206	0.669	0.986
Red blood cell distribution width, %	MF	15.8	15.94	16.03	15.79	0.09	0.838	0.797	0.776
	M	15.77	16.18	15.69	15.79	0.10	0.268	0.121	0.734
	F	15.83	15.68	16.37	15.79	0.16	0.314	0.255	0.366
Platelets, 10^9^/l	MF	362.44	344.68	385.89	342.7	10.76	0.621	0.162	0.935
	M	351.00	356.30	326.90	333.60	15.00	0.456	0.230	0.725
	F	373.89	331.78	451.44	351.80	15.08	0.084	0.014	0.618
Mean platelet volume, fl	MF	9.45	10.09	9.66	9.97	0.12	0.864	0.040	0.884
	M	9.59	10.07	10.01	10.21	0.17	0.422	0.848	0.723
	F	9.30	10.11	9.30	9.72	0.15	0.504	0.041	0.873
Plateletcrit, %	MF	0.34	0.35	0.37	0.34	0.01	0.586	0.586	0.790
	M	0.33	0.36	0.33	0.34	0.02	0.693	0.331	0.852
	F	0.35	0.33	0.43	0.34	0.02	0.216	0.152	0.916
Platelet distribution width, %	MF	36.43	37.55	35.93	37.00	0.23	0.390	0.006	0.988
	M	36.48	37.73	36.21	38.10	0.36	0.943	0.547	0.901
	F	36.38	37.34	35.65	36.48	0.27	0.135	0.093	0.917
Neutrophil to lymphocyte ratio	MF	5.50	5.09	6.16	5.80	0.19	0.077	0.313	0.936
	M	5.08	5.41	6.51	6.24	0.30	0.068	0.030	0.526
	F	5.97	4.74	5.82	5.35	0.23	0.612	0.074	0.416

Both groups had significant changes between timepoints for several hematological parameters.

### Biomarkers

All biomarker data for both the first and final insult are presented in Table 6.

**Table 6.** Biomarker results between undenatured type II collagen (UC-II) and placebo treatment groups (Trx) during the first insult, at timepoints (TP) baseline, pre first 5 km run, and post first 5 km run and during the final insult at timepoints pre 16 km and post 16 km. Biomarkers evaluated were interleukin-6 (IL-6), creatine kinase-MM (CKM), and cartilage oligomeric matrix protein (COMP)

			UC-II			Placebo				*P*-value		
	Variable	Sex	Baseline	Pre 5 km	Post 5 km	Baseline	Pre 5 km	Post 5 km	SEM	Trx	TP	Trx × TP
First Insult	IL-6, ng/ml	MF	0.15^b^	0.14^b^	0.18^a^	0.15^b^	0.17^ab^	0.25^a^	0.01	0.008	0.001	0.198
		M	0.16^ab^	0.14^b^	0.18^a^	0.19	0.22	0.29	0.02	0.013	0.139	0.528
		F	0.14^ab^	0.13^b^	0.17^a^	0.13^b^	0.13^b^	0.21^a^	0.01	0.129	0.001	0.079
	CK, ng/ml	MF	1.69^b^	3.44^a^	3.49^a^	2.09^b^	3.35^ab^	3.91^a^	0.25	0.41	0.001	0.801
		M	1.58^b^	2.85^ab^	4.47^a^	2.21	2.88	3.20	0.34	0.669	0.007	0.265
		F	1.80^b^	4.03^a^	2.40^b^	1.98^b^	3.82^ab^	4.61^a^	0.34	0.136	0.002	0.101
	COMP, ng/ml	MF	43.36^b^	56.50^a^	48.11^ab^	47.49^b^	61.33^a^	54.30^ab^	1.69	0.038	0.001	0.939
		M	41.50	51.10	42.42	45.97^b^	62.28^a^	49.67^ab^	2.66	0.016	0.003	0.672
		F	45.42^b^	61.89^a^	53.80^ab^	49.21	60.48	58.46	2.99	0.514	0.010	0.745
			Pre 16 km	Post 16 km		Pre 16 km		Post 16 km	SEM	Trx	TP	Trx × TP
Final Insult	IL-6, ng/ml	MF	0.25	0.38		0.26		0.43	0.02	0.213	0.001	0.446
		M	0.24	0.39		0.27		0.52	0.03	0.091	0.001	0.279
		F	0.25	0.37		0.25		0.35	0.01	0.333	0.001	0.489
	CK, ng/ml	MF	3.04	4.89		2.17		3.69	0.57	0.204	0.040	0.839
		M	2.93	5.85		2.15		3.76	1.00	0.309	0.112	0.640
		F	3.14	3.93		2.20		3.62	0.63	0.489	0.223	0.721
	COMP, ng/ml	MF	64.01	72.14		71.81		95.36	4.00	0.008	0.007	0.179
		M	54.12	66.63		62.17		78.94	3.69	0.059	0.008	0.685
		F	75.00	79.02		81.45		111.79	6.45	0.039	0.069	0.159

^abc^Within rows and treatment, means without a common superscript are significantly different (*P* < 0.05).

For the first insult, undenatured type II collagen dogs had lower concentrations of IL-6 compared to placebo dogs overall (*P* = 0.008). Undenatured type II collagen dogs had lesser IL-6 at baseline and pre 5 km run compared with post 5 km run, where placebo dogs had elevated IL-6 at pre and post 5 km runs (*P* = 0.001). During the final insult timepoints, IL-6 was lesser at pre 16 km compared with post 16 km for both undenatured type II collagen and placebo groups (*P* = 0.001).

For the first insult, CKM was lower at baseline compared with pre 5 km and post 5 km in undenatured type II collagen dogs (*P* = 0.003). In placebo dogs, baseline was lesser than post 5 km only (*P* = 0.021). Female undenatured type II collagen dogs had lesser CKM compared with placebo females at post 5 km (*P* = 0.039). No significant differences were found between groups or timepoints at the final insult for CKM.

When evaluating COMP, both groups followed similar patterns for the first insult. Baseline values were lesser than the pre 5 km run values for both groups (*P* = 0.001). Both undenatured type II collagen males and females had lesser COMP compared to placebo males and females at all three timepoints (*P* = 0.003; *P* = 0.01). For the final insult, undenatured type II collagen dogs had reduced concentrations of COMP at post 16 km compared with placebo dogs (*P* = 0.023). Placebo dogs also had a significant increase in COMP from pre 16 km to post 16 km (*P* = 0.021), where the change in undenatured type II collagen dogs was not significant (*P* = 0.163). Placebo males had elevated COMP at pre 5 km compared with baseline (*P* = 0.027). Undenatured type II collagen females had lesser COMP at baseline compared with pre 5 km (*P* = 0.027).

## DISCUSSION

Exercise can cause microtrauma in tissue and joints, resulting in a cycle of inflammation and discomfort even in healthy dogs. In a study using hunting dogs, serum concentrations of proinflammatory acute phase proteins serum amyloid A, haptoglobin, and c-reactive protein showed significant elevations both after moderate hunting exercise and in comparison to resting dogs (Casella et al., 2013). Increases in C-reactive proteins, leukocytes, and neutrophils in exercised Spanish Greyhounds after exercise also support the effect of transient subclinical inflammation after strenuous exercise (Lucas et al., 2015).

Endurance running exercise in Labrador Retrievers was used as the inflammatory model in this study, with all dogs participating in a twice weekly endurance running regimen after loading. The first 5 km run and the final 16 km run were used as points of interest for the evaluation of biomarkers. The first 5 km run was considered an insult to unconditioned dogs, and the final 16 km an insult and acute increase in distance to exercise conditioned dogs. Biomarkers of interest for the current experiment included IL-6, CKM, and COMP, as well as a full hematology panel including NLR.

APKm and AMS were monitored during the runs to evaluate exercise performance (Michel & Brown, 2011; Varney et al., 2017). APKm was greater in undenatured type II collagen males, but no significant differences were found in undenatured type II collagen females. AMS, however, was greater overall in undenatured type II collagen dogs compared with placebo. Higher APKm and higher average moving speed can be attributed to improvement in inflammation, pain, and discomfort (Brown et al., 2010; Lascelles et al., 2015), indicating that the undenatured type II collagen supplement improved mobility during the runs.

Hematology was monitored during the experiment. Subsets of white blood cells, including eosinophil and basophil percent, were lesser in undenatured type II collagen dogs compared with placebo dogs after the first 5 km run. Lymphocytes were also greater in undenatured type II collagen dogs compared with placebo dogs at pre 5 km, post 5 km, and post 16 km. However, the placebo groups had a lower lymphocyte count at baseline. Strenuous exercise is associated with increases in white blood cell subsets (Nieman, 1997), with the release of basophils in response to immune challenges and release of eosinophils in response to inflammation. Lower percentages of basophils and eosinophils after the first 5 km run indicate lower inflammation and immune stress in undenatured type II collagen supplemented dogs.

One measure of inflammation is the determination of NLR via hematology analysis (Chandrashekara et al., 2017; Joisten et al., 2019). In the present experiment, the undenatured type II collagen dogs had no significant changes from timepoint to timepoint. However, the placebo dogs had a significant increase in NLR from baseline to pre 5 km, and the placebo dogs had greater NLR at pre 5 km compared with undenatured type II collagen dogs. This may indicate overall lower inflammation in the undenatured type II collagen supplemented dogs.

Proinflammatory cytokine IL-6 is associated with joint inflammation in osteoarthritis (Maccoux et al., 2007; Foster et al., 2014). Circulating IL-6 has been shown to increase in sled dogs during a multiple day race (von Pfeil et al., 2015). In human athletes, immune response to heavy exertion was found to influence cytokines, especially IL-6 (Nieman, 1997). In the present study, both treatment groups had similar baseline measurements of IL-6 and significant increases in IL-6 at the post 5 km timepoint. However, IL-6 was lesser in undenatured type II collagen dogs compared with placebo at post 5 km. Both groups also had a significant increase in IL-6 from pre to post 16 km, and no differences were found between groups. This can likely be attributed to undenatured type II collagen mediating inflammation during normal stressors, but not after a major stressor such as the 16 km run.

Biomarker CKM is associated with skeletal muscle damage (Vlasakova et al, 2017). Both groups had similar patterns of CKM production during the first insult, both developing elevated CKM at both pre and post 5 km compared with baseline. Female undenatured type II collagen dogs had lesser CKM compared with placebo dogs at post 5 km, which may indicate a sex effect. During the final insult, no significant differences between groups were noted. These results indicate the expected response to exercise, but a lack of interaction of undenatured type II collagen supplement on skeletal muscle injury.

Biomarker COMP is associated with cartilage metabolism and degeneration in joint injuries and stress (Saxne & Heinegård, 1992; Misumi et al., 2002). Undenatured type II collagen activates T regulatory cells and releases anti-inflammatory cytokines, reducing joint inflammation and promoting cartilage repair (Gencoglu et al., 2020). In the present study, both groups followed similar patterns, with an increase in COMP from baseline to the pre 5 km timepoint. After the 16 km run, the undenatured type II collagen dogs had lesser COMP compared with placebo dogs. In experiments examining the impact of exercise on COMP, a larger increase in COMP was found in human trials after high impact activities such as box drops compared with low impact activities such as walking. This may explain some of the differences seen after the 5 km run compared with after the 16 km run. Although the dogs were untrained for the first 5 km run, the shorter distance may have prevented cartilage degeneration (Griffin et al., 2011). The longer distance and therefore higher impact of the final 16 km run likely increased cartilage degeneration, allowing differences to be seen between treatment groups.

Limitations of this study include sample collection timing. Blood collection was performed at only one timepoint after exercise based on available literature for the chosen biomarkers, due to budgetary constraints and ethical concerns. Following the biomarkers at key timepoints after the stressor may provide additional insight on the mechanisms of undenatured type II collagen.

In conclusion, inflammation and cartilage degeneration were mitigated during an exercise regimen in undenatured type II collagen supplemented dogs compared with placebo dogs.
